# A comparison of 3- and 4-option multiple-choice items for medical subspecialty in-training examinations

**DOI:** 10.1186/s12909-023-04277-2

**Published:** 2023-04-27

**Authors:** Dandan Chen, Ann E. Harman, Huaping Sun, Tianpeng Ye, Robert R. Gaiser

**Affiliations:** 1 The American Board of Anesthesiology, 4200 Six Forks Road, Suite 1100, Raleigh, NC 27609 USA; 2 Yale School of Medicine, New Haven, CT USA

**Keywords:** 3-option and 4-option multiple-choice items, Psychometric properties of items and exams, Medical subspecialty in-training examinations, Non-functioning distractors

## Abstract

**Background:**

The American Board of Anesthesiology piloted 3-option multiple-choice items (MCIs) for its 2020 administration of 150-item subspecialty in-training examinations for Critical Care Medicine (ITE-CCM) and Pediatric Anesthesiology (ITE-PA). The 3-option MCIs were transformed from their 4-option counterparts, which were administered in 2019, by removing the least effective distractor. The purpose of this study was to compare physician performance, response time, and item and exam characteristics between the 4-option and 3-option exams.

**Methods:**

Independent-samples t-test was used to examine the differences in physician percent-correct score; paired t-test was used to examine the differences in response time and item characteristics. The Kuder and Richardson Formula 20 was used to calculate the reliability of each exam form. Both the traditional (distractor being selected by fewer than 5% of examinees and/or showing a positive correlation with total score) and sliding scale (adjusting the frequency threshold of distractor being chosen by item difficulty) methods were used to identify non-functioning distractors (NFDs).

**Results:**

Physicians who took the 3-option ITE-CCM (mean = 67.7%) scored 2.1 percent correct higher than those who took the 4-option ITE-CCM (65.7%). Accordingly, 3-option ITE-CCM items were significantly easier than their 4-option counterparts. No such differences were found between the 4-option and 3-option ITE-PAs (71.8% versus 71.7%). Item discrimination (4-option ITE-CCM [an average of 0.13], 3-option ITE-CCM [0.12]; 4-option ITE-PA [0.08], 3-option ITE-PA [0.09]) and exam reliability (0.75 and 0.74 for 4- and 3-option ITE-CCMs, respectively; 0.62 and 0.67 for 4-option and 3-option ITE-PAs, respectively) were similar between these two formats for both ITEs. On average, physicians spent 3.4 (55.5 versus 58.9) and 1.3 (46.2 versus 47.5) seconds less per item on 3-option items than 4-option items for ITE-CCM and ITE-PA, respectively. Using the traditional method, the percentage of NFDs dropped from 51.3% in the 4-option ITE-CCM to 37.0% in the 3-option ITE-CCM and from 62.7% to 46.0% for the ITE-PA; using the sliding scale method, the percentage of NFDs dropped from 36.0% to 21.7% for the ITE-CCM and from 44.9% to 27.7% for the ITE-PA.

**Conclusions:**

Three-option MCIs function as robustly as their 4-option counterparts. The efficiency achieved by spending less time on each item poses opportunities to increase content coverage for a fixed testing period. The results should be interpreted in the context of exam content and distribution of examinee abilities.

**Supplementary Information:**

The online version contains supplementary material available at 10.1186/s12909-023-04277-2.

## Introduction

A large and growing body of work has investigated the optimal number of options for multiple-choice items (MCIs) [1–5]. Three-option MCIs have been found to perform as robustly as 4- or 5-option MCIs in K-12 and medical educational settings with minimal changes in item difficulty, item discrimination, and score reliability [4–6]. Reducing the number of options could also provide more efficiency in test administration [6], as many as 16% more MCIs per 1-h testing period [4]. In a fixed testing period, administering more items potentially increases score reliability and content-related validity as more content can be covered. In addition, less time and effort is needed to develop high-quality 3-option MCIs compared to questions with more options [1, 5, 7]. The evidence points to the potential benefits of reducing the number of distractors of 4- or 5-option MCIs for both test takers and test developers.

Despite the empirical evidence and practical rationale favoring 3-option MCIs, the implementation of such practice has been slow, especially in licensure and certification examinations. This could be due to multiple reasons: 1). Traditionally, the 4- or 5- option MCI is the standard format for assessing medical knowledge and clinical skills. Test developers may perceive that reducing the number of options increases the likelihood of guessing and thus threatens the reliability and validity of test scores. Those concerns may be legitimate if all distractors function well (e.g., attract at least 5% of examinees and negatively discriminate). However, more than 90% of questions on medical exams have at least one distractor that attracts fewer than 5% of examinees [8, 9]. 2). There is a paucity of research on licensure and certification exams to support the development and implementation of 3-option MCIs in the national setting. Previous studies directly comparing the psychometric properties of 3- and 4-option MCIs were mostly performed in local medical schools for summative assessment [9]. Only 7 out of 56 empirical studies from 1925 to 1999 included in Rodriguez’s meta-analysis were related to professional exams [1]. There are concerns about the potential to jeopardize the process of making sound high-stakes decisions about candidates (e.g., determining whether physicians be certified in a medical specialty). 3). In the last decade, the psychometric properties of a mix of 3-, 4-, and 5-option MCIs in the same test form were investigated [7], but there were no direct comparisons among different numbers of MCI options as the entire test form.

To close the gap between research and implementation of 3-option MCIs in medical exams, the American Board of Anesthesiology (ABA, Raleigh, NC) piloted 3-option single-best-answer, multiple-choice questions in two anesthesiology subspecialty in-training examinations (ITEs) – all the 3-option questions were transformed from their previously administered 4-option counterparts by removing the least effective distractor. The purpose of this study was to compare physician performance, response time, and item and exam characteristics between the 4-option and 3-option formats of the otherwise same exam form. Considering the findings in previous research [2, 9, 10], we hypothesized that physicians’ percent-correct scores would be slightly higher, and physicians would spend less time per item on 3-option ITEs in comparison with 4-option ITEs. Based on the findings by Rodriguez [1], we predicted a slight decrease in item difficulty and similar item discrimination as well as exam reliability when the number of options reduced from 4 to 3. In addition, we aimed to assess whether 3-option MCIs would have lower percentages of non-functioning distractors (NFDs) and higher percentages of items without NFDs than their 4-option counterparts.

## Methods

This study was determined to be exempt from review by WCG Institutional Review Board (Puyallup, WA). Informed consent was obtained from the physicians when they registered for the ABA examinations by signing an Acknowledgement and Release to allow their information to be used for research. De-identified physician item data were used for the analyses.

### Examination construction

The ABA offers subspecialty ITEs for physicians who have completed anesthesiology residency and are currently enrolled in subspecialty fellowship training. The ITEs are designed to evaluate fellows’ progress toward meeting the educational objectives of subspecialty training and share the same content outlines and item pools with the subspecialty certification exams. Although no pass-fail decisions are made, ITEs serve as a knowledge check-in for fellows in training and the vast majority of them will sit for the subspecialty certification exams in a few months. The ABA used 5-option MCIs as one of its item types before 2010 and transitioned to 4-option MCIs for its written exams in the early 2010s. In 2020, 3-option MCIs were piloted for ITEs for Critical Care Medicine (ITE-CCM) and Pediatric Anesthesiology (ITE-PA).

The 3-option items of the ITE-CCM and ITE-PA were derived from 4-option items by two approaches. For items in which there was a distractor chosen by no examinees [*n*_*i*_ = 14 (9.3% of the total 150 items) for ITE-CCM and *n*_*i*_ = 44 (29.3% of the total 150 items) for ITE-PA], that distractor was automatically removed. For the rest of the items, an examination committee (consisting of a half dozen subject matter experts) determined which distractor should be removed based on their best judgment; distractor analyses, including percentage of examinees choosing each option and correlations between choosing each option and total score, based on 4-option items were available to them. Item stems (*n*_*i*_ = 150) were identical for both the 2019 (4-option) and 2020 (3-option) administrations of the ITE-CCM and ITE-PA.

### Analytic strategies

For the first set of analyses relating to physician performance, independent-samples t-tests were used to examine the differences in the physician’s percent-correct scores between 4-option and 3-option ITEs, which refer to the percentage of items on the exam answered correctly by each physician; paired t-tests were used to examine the differences in response time per item (the average number of seconds that physicians spent per item) and response speed (the average number of seconds spent per word, with item word count including both the stem and the options) between the 4-option and 3-option formats.

The second set of analyses used paired t-tests to examine the differences in item difficulty and item discrimination between the 4-option and 3-option ITEs. According to the Classical Test Theory, in which item characteristics are bound with a particular test and an examinee sample [11], item difficulty or *p-value* is defined as the proportion of physicians taking the exam who answered the question correctly; item discrimination is the corrected point-biserial correlation (*cRpb*) between the item correctness and the total score based on the rest of the items on the exam (i.e., excluding the item itself) [12]. The Kuder and Richardson Formula 20 (KR-20) [13] was used to calculate the reliability of each form of the ITEs.

The third set of analyses focused on the differences in NFDs between 3- and 4-option MCIs as determined by both the traditional and sliding scale methods. Traditionally, NFDs are defined as distractors selected by fewer than 5% of examinees and/or showing a positive correlation with total score on a test [1, 7, 10]. The sliding scale method builds on the previous definition of NFD and  defines the distractor selection threshold conditionally on an item’s difficulty such that the easier the item, the lower the threshold of the percentage of examinees choosing a distractor used to identify it as non-functioning. The specification of this sliding scale method is illustrated in the equation below [7]:

$${p}_{nfd}=0.1-({p}_{c}*0.1)$$

where ${p}_{c}$ is the proportion of examinees choosing the correct answer. A distractor is flagged as nonfunctional if the proportion of examinees choosing this distractor is lower than ${p}_{nfd}$. For both traditional and sliding scale methods, we also considered a positive point-biserial correlation between the distractor and the total score as a criterion for flagging NFDs. The count and percentage of NFDs were reported for each exam administration. The number of NFDs per item was also reported to compare the efficacy of distractors on 3- and 4-option MCIs. All the statistical analyses were conducted in R 4.2.0 (Vienna, Austria).

## Results

### Physician performance

Physicians who took 3-option MCIs had higher percent-correct scores than those who took 4-option MCIs for the ITE-CCM (67.7 ± 6.7 versus 65.7 ± 6.9, Table 1), *t*_(273)_ = 2.514, *p* = 0.013, with a small-to-medium effect size (Cohen’s *d* = 0.30) [14]. However, for the ITE-PA, there was no significant difference in physician percent-correct scores, *t*_(243)_ = 0.102, *p* = 0.919. Overall the ITE-CCM was a more difficult exam than the ITE-PA (6.1% correct lower for the 3-option MCIs, and 4.0% lower for the 4-option MCIs).

**Table 1:** Physician performance and item characteristics for 3- and 4-option MCI ITEs

	**Percent-correct Score, Mean ± SD**	**Response time (seconds per item), Mean **$\pm$** SD**	**Response speed (seconds per word), Mean **$\pm$** SD**	**Item difficulty, Mean **$\pm$** SD**	**Item discrimination, Mean **$\pm$** SD**
**ITE-CCM**					
4-option	65.7 $\pm$ 6.9	58.9 $\pm$ 22.8	0.98 $\pm$ 0.53	0.66 $\pm$ 0.21	0.13 $\pm$ 0.09
3-option	67.7 $\pm$ 6.7	55.5 $\pm$ 20.8	0.97 $\pm$ 0.51	0.68 $\pm$ 0.20	0.12 $\pm$ 0.10
Mean difference	2.1*	-3.4***	-0.01	0.02***	-0.01
**ITE-PA**					
4-option	71.8 $\pm$ 5.0	47.5 $\pm$ 19.0	1.19 $\pm$ 0.44	0.72 $\pm$ 0.25	0.08 $\pm$ 0.11
3-option	71.7 $\pm$ 5.4	46.2 $\pm$ 18.4	1.25 $\pm$ 0.45	0.72 $\pm$ 0.24	0.09 $\pm$ 0.11
Mean difference	-0.1	-1.3**	0.06 ***	0	0.02

For ITE-CCM, *n*_*p2019*_ = 152 in 4-option and *n*_*p2020*_ = 123 in 3-option format; for ITE-PA, *n*_*p2019*_ = 113 in 4-option and *n*_*p2020*_ = 132 in 3-option format

^*^ indicates *0.01* $<$ *p* ≤ *0.05*; ** indicates *0.001* $<$ *p* ≤ *0.01*; *** indicates *p* ≤ *0.001*

Although response time per item was moderately correlated with item word count for both formats of the ITE-CCM [r = 0.64 (95% CI, 0.54 to 0.73) for 4-option items; r = 0.65 (95% CI, 0.55 to 0.74) for 3-option items] and ITE-PA [r = 0.50 (95% CI, 0.37 to 0.61) for 4-option items; r = 0.53 (95% CI, 0.40 to 0.63) for 3-option items], response speed (seconds per word) and response time per item showed slightly different patterns for the two exams (Table 1). For the ITE-CCM, the response speed was not statistically significant between 4-option and 3-option formats, *t*_(149)_ = 1.060, *p* = 0.330, and the 3-option items took less time than the 4-option items, *t*_(149)_ = 7.720, *p* < 0.001, with a medium-to-large effect size (Cohen’s *d* = 0.63) [14]. For the ITE-PA, the response speed was significantly slower for 3-option than 4-option items, *t*_(149)_ = 4.394, *p* < 0.001, with a small-to-medium effect size (Cohen’s *d* = 0.36) [14], and 3-option items still took less time to answer than 4-option items, *t*_(149)_ = -2.880, *p* = 0.005, with a small effect size (Cohen’s *d* = 0.24) [14]. On average, physicians who took ITE-CCM and ITE-PA spent 3.4 s (55.5 ± 20.8 versus 58.9 ± 22.8) and 1.3 s (46.2 ± 18.4 versus 47.5 ± 19.0) less per item for 3-option than 4-option MCIs, respectively.

### Item and examination characteristics

For the ITE-CCM, there was a statistically significant difference in item difficulty [i.e., *p-values*; *t*_(149)_ = 4.025, *p* < 0.001, (Table 1)]. The effective size was small to medium (Cohen’s *d* = 0.33) [14], with 3-option MCIs being slightly easier (i.e., 2% higher) than 4-option MCIs. Item discrimination difference was not significant [i.e., *cRpb*, *t*_(149)_ = 0.687, *p* = 0.493] between 4-option and 3-option MCIs. For the ITE-PA, no statistically significant difference was found in item difficulty [*t*_(149)_ = 0.125, *p* = 0.901] or item discrimination [*t*_(149)_ = 1.372, *p* = 0.172] between 4-option and 3-option MCIs.

For the ITE-CCM, the KR-20 equaled 0.75 for 4-option and 0.74 for 3-option MCIs. For the ITE-PA, the KR-20 equaled 0.62 for 4-option and 0.67 for 3-option MCIs.

### Distractor functionality

Three-option MCIs tended to have lower percentages of NFDs than 4-option MCIs regardless of flagging criteria or exam subspecialty (Table 2). For the ITE-CCM, the percentage of NFDs dropped from 51.3% to 37.0% using the traditional “frequency and/or discrimination” method, and from 36.0% to 21.7% using the sliding scale “frequency and/or discrimination” method. For the ITE-PA, the percentage of NFDs changed from 62.7% to 46.0% using the traditional “frequency and/or discrimination” method, and from 44.9% to 27.7% using the sliding scale “frequency and/or discrimination” method. The results showed similar patterns for frequency alone or discrimination alone criterion, and the reduction in the percentage of NFDs was greater for the frequency alone than the discrimination alone criterion.

**Table 2:** Number and percent of nonfunctioning distractors for 3- and 4-option MCI ITEs

	ITE-CCM				ITE-PA			
	2019, 4-opt		2020, 3-opt		2019, 4-opt		2020, 3-opt	
	n	%	n	%	n	%	n	%
**Traditional method**								
Frequency	188	41.8	77	25.7	241	53.6	107	35.7
Discrimination	82	18.2	46	15.3	89	19.8	52	17.3
Frequency and/or discrimination	231	51.3	111	37.0	282	62.7	138	46.0
**Sliding scale method**								
Frequency	101	22.4	24	8.0	132	29.3	35	11.7
Discrimination	82	18.2	46	15.3	89	19.8	52	17.3
Frequency and/or discrimination	162	36.0	65	21.7	202	44.9	83	27.7

Total distractors equal to 450 for 4-option MCI ITEs and 300 for 3-option MCI ITEs

Using the traditional method, the number of items without NFDs increased from 12 to 58 (8.0% to 38.7%) for the ITE-CCM and 6 to 45 (4.0% to 30.0%) for the ITE-PA after converting the MCIs from 4- to 3-option format (Table 3). Using the sliding scale method, the number of items without NFDs increased from 27 to 88 (18.0% to 58.7%) for the ITE-CCM and from 19 to 78 (12.7% to 52.0%) for the ITE-PA after converting the MCIs from 4- to 3-option format.

**Table 3:** Number and percent of items with nonfunctioning distractors for 3- and 4-option MCI ITEs

	ITE-CCM				ITE-PA			
	2019, 4-opt		2020, 3-opt		2019, 4-opt		2020, 3-opt	
	n	%	n	%	n	%	n	%
**Traditional method**								
0	12	8.0	58	38.7	6	4.0	45	30.0
1	65	43.3	73	48.7	42	28.0	72	48.0
2	53	35.3	19	12.7	66	44.0	33	22.0
3	20	13.3	-	-	36	24.0	-	-
*Total*	*150*	*100*	*150*	*100*	*150*	*100*	*150*	*100*
**Sliding scale method**								
0	27	18.0	88	58.7	19	12.7	78	52.0
1	86	57.3	59	39.3	68	45.3	61	40.7
2	35	23.3	3	2.0	55	36.7	11	7.3
3	2	1.3	-	-	8	5.3	-	-
*Total*	*150*	*100*	*150*	*100*	*150*	*100*	*150*	*100*

## Discussion

Our findings are consistent with the literature that there were minimal changes in physician performance and psychometric properties when changing 4- to 3-option MCIs [1, 4, 9, 10, 15]. Because no physicians chose one of its distractors, 9.3% of 4-option ITE-CCM items and 29.3% of 4-option ITE-PA items were de facto 3-option MCIs; the proportion of such items were likely higher among high-performing examinees as they tended to eliminate most obvious distractors and reduce their choices to one or two options. It is not surprising that the changes in physician percent-correct score and item difficulty were statistically significant for the ITE-CCM only, with a small-to-medium effect size. Agreeing with the literature [5, 7, 9], the results of distractor analyses for both subspecialty ITEs showed that the percentage of NFDs across all methods and criteria decreased and the percentage of items without any NFDs increased when the number of MCI options was reduced. This provides supportive evidence that 3-option MCIs are sufficient to distract examinees with lower overall ability and that the 4-option MCIs may be too “fat” to provide any additional meaningful information [15]. Therefore, these findings point to the feasibility of transitioning to 3-option MCIs for medical subspecialty certification exams.

Consistent with previous studies [4], physicians took less time to answer 3-option than 4-option MCIs for both subspecialty ITEs. On average, physicians’ response time to ITE-CCM items was longer than ITE-PA items, and response speed for 3-option ITE-PA items was actually slower than for 4-option ITE-PA items. The average word count per item was 68.4, 65.7, 42.4, and 39.2 for 4-option ITE-CCM, 3-option ITE-CCM, 4-option ITE-PA, and 3-option ITE-PA, respectively (Supplement Material 1 – sample questions for Critical Care Medicine and Pediatric Anesthesiology). Physicians seemed to have been required to obtain more information from a clinical vignette of a CCM item to make a diagnosis or a clinical judgment than a PA item, which was reflected in the fact that CCM items were longer than PA items and physicians spent about 10 more seconds per item on the ITE-CCM than the ITE-PA. In addition, the ITE-CCM included 50 images or tables and the ITE-PA included 9 images or tables, which were not reflected in the word count. Medical educators and test developers need to be aware that reducing the number of options per MCI may not necessarily save substantial testing time.

The change in the score reliability associated with the change in number of the options varies, depending on the option deletion method and other factors [1, 9, 10]. In general, if the least effective distractor is properly identified and removed from MCIs, the score reliability is expected to increase [16]. In this study, the reliability coefficient of the ITE-PA increased by 0.05 and yet the reliability coefficient of the ITE-CCM decreased by 0.01 after converting the 4-option questions to 3-option by removing the least effective distractor. Although the magnitude of these changes was minimal, it is worth noting that reducing the number of distractors does not necessarily positively impact the reliability of test scores. Reliability reflects how well the items on a test can consistently distinguish examinees with a range of abilities. The reason we found a minimal change of reliability coefficients may be the presence of a restricted range of physicians’ performance [17]. Only physicians enrolled in subspecialty fellowship training after completing a residency were eligible to take the exams. In general, physicians who are more motivated to specialize their clinical practice areas or more competent in medical knowledge and clinical skills are more likely to be admitted to fellowship programs. Also, the nature of the subspecialty and exam content may have played a role. The ITE-CCM was, overall, a more difficult and discriminative exam than the ITE-PA.

MCI distractors do not function equally well. Previous studies have shown that more than 90% of MCIs on medical exams have at least one distractor that attracts fewer than 5% of examinees [8, 9]. The distractor analyses in this study further support the conclusion that the quality of distractors matters more than the quantity [15]. Anecdotally, our question authors are very pleased with not having to come up with a 3^rd^ distractor when constructing 3-option MCIs, which typically take disproportional amount of time compared to two distractors. We expect financial cost of developing 3-option MCIs will go down as the efficiency of developing such items increases. Although not having to create a 3^rd^ distractor is desirable for question authors and test developers, it becomes more critical for 3-option MCIs that each distractor functions effectively (e.g., representing common misconceptions or errors in thinking and reasoning among lower-ability examinees). Training question authors in a systematic way, such as use of concept mapping and the crafting of realistic clinical scenarios, is essential for producing effective distractors to ensure successful implementation of 3-option MCIs [7]. In additional to the time and financial efficiency expected to be achieved by 3-option MCIs, these approaches could be particularly useful in the formative assessments to gauge learners’ understanding of important concepts and design instructions accordingly to clear common misconceptions or reasoning errors. Conversely, more advanced learners may appreciate fewer but higher-quality distractors as such distractors would promote more advanced reflective thinking of why they are plausible but not the correct answer.

This study was subject to limitations. First, the 4-option ITEs were administered in spring 2019 (prior to the Covid-19 pandemic), and the 3-option ITEs were administered in spring 2020 during the pandemic, which may affect the level of stress felt by the examinees. Second, our approach to the elimination of distractors was conservative in that distractors not chosen by any examinees were deleted first and then subject matter experts made their best judgment of which distractors should be deleted from other items. Using the sliding scale method to identify NFDs may accelerate the process of transforming 4-option to 3-option MCIs. Finally, no pass/fail decisions were made based on the ITEs reported in this study. Future studies should investigate how to set fair and defensible standard(s) for exams with 3-option MCIs if any definitive decisions have to be made about the examinees. The overall guess rate of the 3-option MCIs is naturally 33%, higher than the guess rate of 25% of 4-option MCIs. In contrast to the Classical Test Theory, the Item Response Theory estimates item parameters independently from the examinee samples [18]. When the sample size is large enough to achieve accurate parameter estimates (e.g., at least a thousand examinees for a 150-item exam [19], the 3-parameter Item Response Theory model could account for guess rate at the item level, in addition to item difficulty and item discrimination, which would help maintain the standard(s) more objectively.

In conclusion, this study extends previous evidence that 3-option MCIs function as robustly as their 4-option counterparts in item difficulty and item discrimination [4, 9, 10, 20] to the subspecialty ITEs offered by a medical specialty certifying board. Furthermore, the exam content and the distribution of examinee abilities may play an important role in the physician performance, response speed, response time, and psychometric properties of items and exams. Both quantitative indices and qualitative judgement from subject matter experts could contribute to identifying or revising ineffective distractors.

## Supplementary Information

**Additional file 1.** Supplemental Material 1 - sample questions for Critical Care Medicine and Pediatric Anesthesiology.

## Data Availability

The datasets generated and/or analyzed during the current study are not publicly available due to the confidentiality and sensitivity of the examination data. De-identified datasets may be available from the corresponding author on reasonable request, and are subject to a data sharing mandate.
