# Supplementary material for: A comparison of 3- and 4-option multiple-choice items for medical subspecialty in-training examinations
Source: BMC Med Educ. 2023 Apr 27;23:286. doi: 10.1186/s12909-023-04277-2 (PMC10134669; doi:10.1186/s12909-023-04277-2)
Supplement: Supplementary file 1 — Additional file 1. Supplemental Material 1 - sample questions for Critical Care Medicine and Pediatric Anesthesiology. [file 12909_2023_4277_MOESM1_ESM.docx]

Supplemental Material 1.

*a. Sample Question for Critical Care Medicine – Distractor C was removed for the 3-option version because it was least chosen by candidates, with a positive discrimination index (the asterisk indicates the correct answer).*

A 39-year-old woman is brought to the ED by ambulance after being found unconscious. She has a history of major depressive disorder. Temperature is 37°C, blood pressure is 130/70 mmHg, heart rate is 120 bpm, and respiratory rate is 22/min. The patient is comatose, and her pupils are fixed and dilated. Glasgow coma scale score is 5. Laboratory studies of serum show:


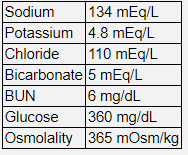


Arterial blood gas analysis shows:


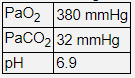


Measurement of which of the following serum concentrations is the **MOST** appropriate **NEXT** step to confirm the diagnosis?

Options:

A.  Beta-hydroxybutyrate

*B.  Ethylene glycol

C.  Lactate

D.  Salicylate

*b. Sample question for Pediatric Anesthesiology* – *Distractor A was removed for the 3-option version based on subject matter experts’ best judgement because all 3 distractors attracted similar number of candidates, with negative discrimination indices.*

A 5-year-old girl requires a craniotomy for subarachnoid hemorrhage following a motor vehicle collision. Which of the following parameters is consistent with a postoperative diagnosis of syndrome of inappropriate antidiuretic hormone secretion (SIADH)?

Options:

A.  Urine-specific gravity < 1.005

B.  Serum osmolality 300 mOsm/L

*C.  Serum sodium 128 mEq/L

D.  Urine sodium < 20 mmol/L
